# Rare Presentation of Immunoglobulin G4–Related Disease: Hepatic Mass Lesions Mimicking Metastasis

**DOI:** 10.5152/tjg.2025.25211

**Published:** 2025-09-18

**Authors:** Tuba Yılmaz Yıldırım, Uğur Çiftçi, Javid Huseynov, Coşkun Özer Demirtaş, Osman Cavit Özdoğan

**Affiliations:** Division of Gastroenterology and Hepatology, Marmara University School of Medicine, İstanbul, Türkiye

Dear Editor,

Immunoglobulin G4 (IgG4)–related disease (IgG4-RD) is a fibrous inflammatory condition that involves multiple organs such as the liver, pancreas, kidney, lymph nodes, lungs, and salivary glands. It is characterized by the infiltration of IgG4-positive plasma cells, development of mass-forming lesions, and increased serum IgG4 levels.^1^ In this reporta rare disease of IgG4-related inflammatory pseudotumors involving the liver has been presented. Radiological, clinical, and pathological outcomes of the patient were analyzed

A 56-year-old male patient presented with right upper quadrant pain, fever, jaundice, nausea, and weakness for 2 days and was admitted to the hospital with a preliminary diagnosis of cholangitis. Informed consent was obtained from the patient. He had no chronic disease, and his medical and family history were unremarkable. In his etiological evaluation, he describes a history of subtotal cholecystectomy and stent placement in the common bile duct due to choledocholithiasis three years ago. Laboratory tests was showed elevated leukocyte level (12 840/mm3, reference range: 4000-10 000/mm3), transaminase, cholestatic level [ALT (Alanine aminotransferase): 217 U/L (reference range: 7-50 U/L), AST (Aspartate aminotransferase): 103 U/L (normal range: 8-50 U/L), GGT (Gamma-glutamil transferase): 243 U/L (reference range: 0-55 U/L), ALP (Alkaline phosphatase): 151 U/L (normal range: 43-115U/L), TB (Total Bilirubin)/DB (Direct Bilirubin): 4.8/2.8 mg/dL (reference range: 0.3-1.2; 0-0.2 mg/dL respectively )], INR (International normalized ratio): 1.1, CRP (C-Reaktive protein): 222 mg/L (reference range: 0-5 mg/L) and procalcitonin 6.6 μg/L (0-0.5 ug/L), IgG4: 1.84 ( reference range: 0.03-2.01 g/L), AFP (ALpha-fetoprotein):2. Computed tomography scans showed bile duct ve intrahepatic dilatation, inflamated bile duct (cholangitis?) and multiple hypoechoic nodules in liver (Figure 1). Magnetic resonance cholangiography showed a gallbladder stone (2.5 cm), wall thickness (5 mm), and dilatation in the intrahepatic and extrahepatic bile duct (11 mm) that was surrounded by edema, T2 hyperintense lesions in the liver parenchyma that were initially thought to be hepatic metastases. Endoscopic retrograde cholangiopancreaticography (ERCP) was performed. The bile duct wall was normal, and there was no stricture. The occluded stent was removed, and the stone was extracted from the bile duct. A 10 cm 10F plastic stent was replaced in the bile duct. Levels of acute phase reactants, transaminase, and cholestatic enzymes were decreased after ERCP and antibiotic treatment. For the investigation of any malignancy, upper and lower gastrointestinal endoscopy was performed, but neither showed any pathological findings. Primovist contrast dynamic liver-specific magnetic resonance imaging (MRI) showed hyperintense, scattered, confluent lesions in the liver parenchyma after IVCM (Intravenous contrast media), which were supportive of metastases. Positron emission tomography (PET)/CT indicated increased FDG (Fluorodeoksiglukoz)-uptake in metastatic-like lesions in the liver (the largest one 2.5 cm in size with a SUVmax of 6.1) without any other pathological findings (Figure 2). The metastatic liver lesions were biopsied, and the histological evaluation revealed fibrosis, chronic inflammatory cells that were rich in plasma and lymphoid cells, occasionally including eosinophil leukocytes, and an IgG4/IG ratio of approximately 30%-35% (Figure 3). Patient was diagnosed with IgG4-related hepatic pseudotumors, without the involvement of the biliary tract and pancreas. Afterwards, 32 mg methylprednisolone treatment was started. One month later, the pseudotumoral lesions disappeared in the follow-up MRI (Figure 4). Methylprednisolone treatment was tapered with the intention of providing 4 mg low dose for 6 months.

IgG4-related disease affects both genders, but mostly middle-aged to elderly men. It is a chronic inflammatory disorder characterized by fibrosis affecting multiple digestive organs. These diseases are identified by the presence of IgG4-positive plasma cell infiltration with fibrosis, elevated serum levels of IgG4, obliterative phlebitis, and eosinophilic infiltration.^1^^,^^2^ Hepatic pseudotumors are rare lesions that mimic malignant lesions radiologically and clinically. Hepatic pseudotumors respond well to conservative treatment with steroids.^3^ Patients usually have subacute enlarged masses in the affected organs.^1^IgG4-related disease is a recently recognized disease characterized by elevated serum IgG4 concentration, lymphoplasmacytic tissue infiltration by IgG4-positive plasma cells, and tissue fibrosis. Although serum IgG4 level is included in the diagnostic criteria, about 3%-30% of IgG4-related disease patients have normal serum IgG4 concentrations.^4^ The 2020 Japanese revised comprehensive criteria consist of 3 items; 1) Clinical and radiological finding indicate that 1 or more organs have localized or diffuse mass characteristic of IgG4-RD, 2) Serological diagnosis with serum IgG4 level above 135 mg/dL, 3) Pathological diagnosis that 2 of 3 criteria; plasma cell and lymphocyte infiltration, tissue fibrosis, IgG4/IgG ratio above 40%. (1) and (3) were diagnosed with probable IgG4-RD.^5^

In this case, IgG4/IgG ratio was approximately 30%-35% but serum IgG4 level was normal. In histology, IgG4-positive plasma cells have been suggested, ranging from 10 to 50. Computed tomography shows nodular lesions as inflammatory pseudotumors. In this case, probable IgG4-RD was considered and steroid treatment was applied. There was a response to treatment, and nodular lesions disappeared.

## Figures and Tables

**Figure 1.**
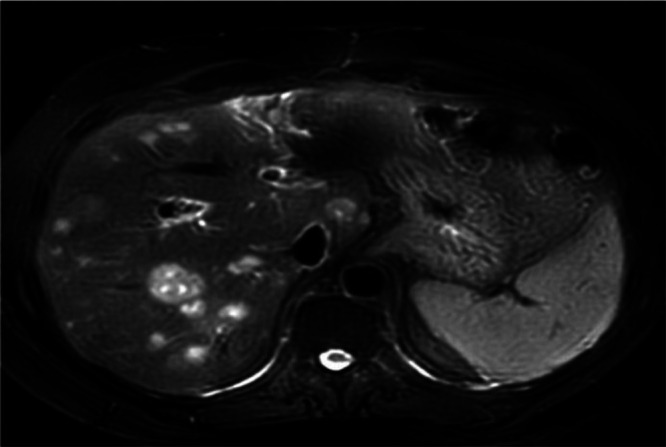
Computed tomography scans showing multiple hypoattenuation nodules.

**Figure 2.**
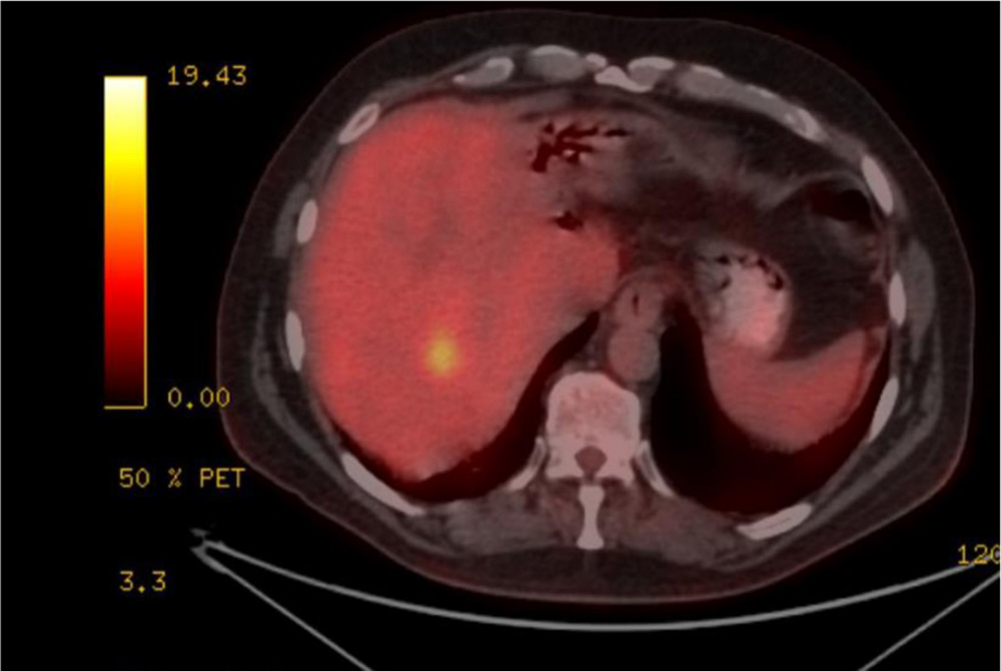
Positron emission tomography/computed tomography indicated FDG-uptake that metastatic-like lesions in the liver.

**Figure 3.**
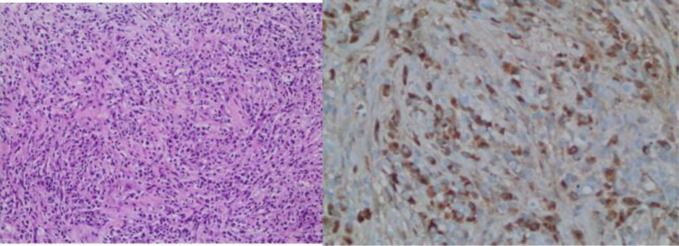
Lymphoplasmacytic cell infiltration and Immunoglobulin G4 staining in liver biopsy.

**Figure 4.**
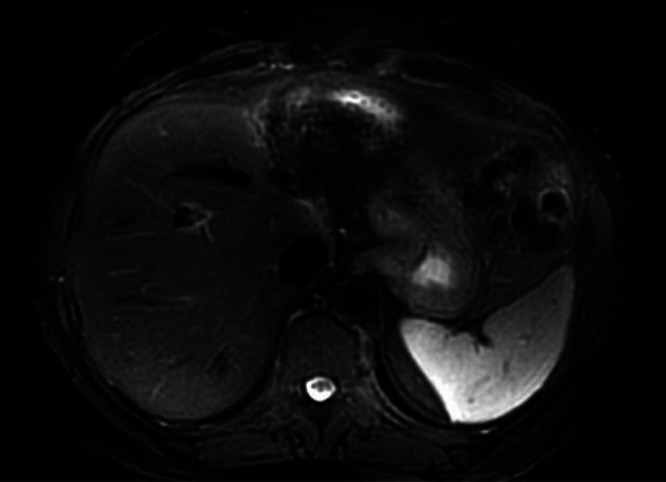
One month after treatment, magnetic resonance imaging shows disappear of pseudotumoral lesions on liver.

## Data Availability

The data that support the findings of this study are available on request from the corresponding author.
